# Analyzing aberrant DNA methylation in Colorectal cancer uncovered intangible heterogeneity of gene effects in the survival time of patients

**DOI:** 10.21203/rs.3.rs-2957915/v1

**Published:** 2023-05-29

**Authors:** Saeedeh Hajebi Khaniki, Farhad Shokoohi, Habibollah Esmaily, Mohammad Amin Kerachian

**Affiliations:** 1 Department of Biostatistics, School of Health, Mashhad University of Medical Sciences, Mashhad, Iran; 2 Department of Mathematical Sciences, University of Nevada-Las Vegas, Las Vegas, NV 89154, USA; 3 Social Determinants of Health Research Center, Mashhad University of Medical Sciences, Mashhad, Iran; 4 Medical Genetics Research Center, Mashhad University of Medical Sciences, Mashhad, Iran

## Abstract

Colorectal cancer (CRC) involves epigenetic alterations. Irregular gene-methylation alteration causes and advances CRC tumor growth. Detecting differentially methylated genes (DMGs) in CRC and patient survival time paves the way to early cancer detection and prognosis. However, CRC data including survival times are heterogeneous. Almost all studies tend to ignore the heterogeneity of DMG effects on survival. To this end, we utilized a sparse estimation method in the finite mixture of accelerated failure time (AFT) regression models to capture such heterogeneity. We analyzed a dataset of CRC and normal colon tissues and identified 3,406 DMGs. Analysis of overlapped DMGs with several Gene Expression Omnibus datasets led to 917 hypo- and 654 hyper-methylated DMGs. CRC pathways were revealed via gene ontology enrichment. Hub genes were selected based on Protein-Protein-Interaction network including *SEMA7A*, *GATA4*, *LHX2*, *SOST*, and *CTLA4*, regulating the Wnt signaling pathway. The relationship between identified DMGs/hub genes and patient survival time uncovered a two-component mixture of AFT regression model. The genes *NMNAT2*, *ZFP42*, *NPAS2*, *MYLK3*, *NUDT13*, *KIRREL3*, and *FKBP6* and hub genes *SOST*, *NFATC1*, and *TLE4* were associated with survival time in the most aggressive form of the disease that can serve as potential diagnostic targets for early CRC detection.

## 1 Introduction

Colorectal cancer (CRC), the third most common cancer worldwide, is a group of diseases characterized by genetic and epigenetic changes^1,2^. Despite being the second leading cause of cancer-related deaths, less attention has been paid to early detection due to the fact that patients do not adhere to invasive screening tests such as colonoscopy^3^. It has been shown that epigenetic alterations in solid and liquid biopsies can be used for early detection and thus prognosis and effective treatment^4^. DNA methylation at CpG sites (5mc) is an epigenetic mark that regulates gene expression through transcriptional silencing^5^. Aberrant DNA methylation plays a crucial role in the pathogenesis and progression of CRC and has emerged as a promising diagnostic marker for the disease^6^. In particular, aberrant DNA methylation can impact genes where their inactivation may exacerbate tumor formation through the induction of genomic instability or by directly silencing the methylated gene^7^.

Much research has been done to develop comprehensive panels of biomarkers based on DNA methylation that can facilitate accurate diagnosis of CRC^8^. While the genes *SEPT9, NDRG4*, and *BMP3* are FDA-approved for CRC^9,10^, there are many other genes such as *APC, SFRP1, TFPI2*, and *VIM* that have not yet been approved^8^.

In order to detect and validate genes that are potential CRC biomarkers, the following steps should be taken. Firstly, a panel of biomarkers must be developed using accurate statistical methods with a deep understanding of the underlying biology of the disease and the molecular mechanisms that drive them. Secondly, the significant biomarkers must be validated via in silico validation using several other datasets; and thirdly, the effectiveness of top candidate biomarkers in improving patient health should be verified using survival models. Lack of adequate precision in each of the above steps leads to misleading conclusions. Among others, two issues affect precision: removing genomic positions with missing values or low read-depth and ignoring the heterogeneity of DMG effects on survival times.

To accurately predict the differentially methylated profiles in CRC, one must consider all biological and environmental factors such as dietary^11^, aging^12^, and hazardous behaviors^13^ (e.g., smoking), among others. Such factors are often ignored by most studies when predicting methylation profiles. In addition, methylation data always suffer from heavy missing values that can affect subsequent analyses. For instance, 68% of CpG sites have missing values in at least one sample in our dataset (Section 2). Almost all DNA methylation pipelines, except a few such as the DMCHMM method^14^, filter out such positions from the analysis. We used DMCHMM to not only account for extra covariates but also efficiently impute the missing values.

Having identified the differentially methylated genes (DMG) associated with CRC and validating them, it is crucial to identify their underlying signaling pathways that regulate gene expression^15,16^. The main known CRC pathways are Wnt^17^, MAPK^18^, TGF-*β*^19^, and TP53^20^. Although significant progress has been made in understanding the biology of CRC, there are still many unknown pathways and mechanisms involved in this disease. Identification of hub genes, also known as driver genes is the next step in the analysis of biomarker detection. Hub genes play a critical role in regulating several genes in the biological network and have the potential to be regarded as therapeutic targets in CRC^21^.

In the next step, the relationship between identified DMGs and the survival time of CRC patients should be evaluated. Most studies employ a limited panel of biomarkers selected through conventional univariate Cox proportional hazard regression models and overlook the potential effects of the rest of the biomarkers^22–24^. In a recent study^25^, the Cox-LASSO survival model was used to account for a larger set of biomarkers but ignored the heterogeneity of covariate effects. To the best of our knowledge, none of the studies have taken into account the heterogeneity of DMG effects on survival time. To address this problem, one may use the sparse estimation method in the finite mixture of accelerated failure time (AFT) regression models^26^. Prior to this step, it is common to screen the number of genes to a manageable magnitude. This process can be done by selecting the top highly correlated genes with survival time of the patients using the correlation-adjusted scoring method^27^.

This study aimed to identify CRC-related DMGs to serve as potential biomarkers for early detection by including all the available information in the data and avoiding the exclusion of any genomic position. To this end, we acquired a high-throughput DNA methylation dataset which consists of patients with CRC and healthy individuals. Information on age, history of smoking, and drug abuse was also collected. A description of the data is provided in Section 2. Information on other datasets used for validation and survival analysis and all statistical and Bioinformatics methods are listed in this section. In Section 3, a comprehensive analysis of data is conducted. Section 4 gives a discussion and some concluding remarks.

## 2 Methods

**Figure 1.**
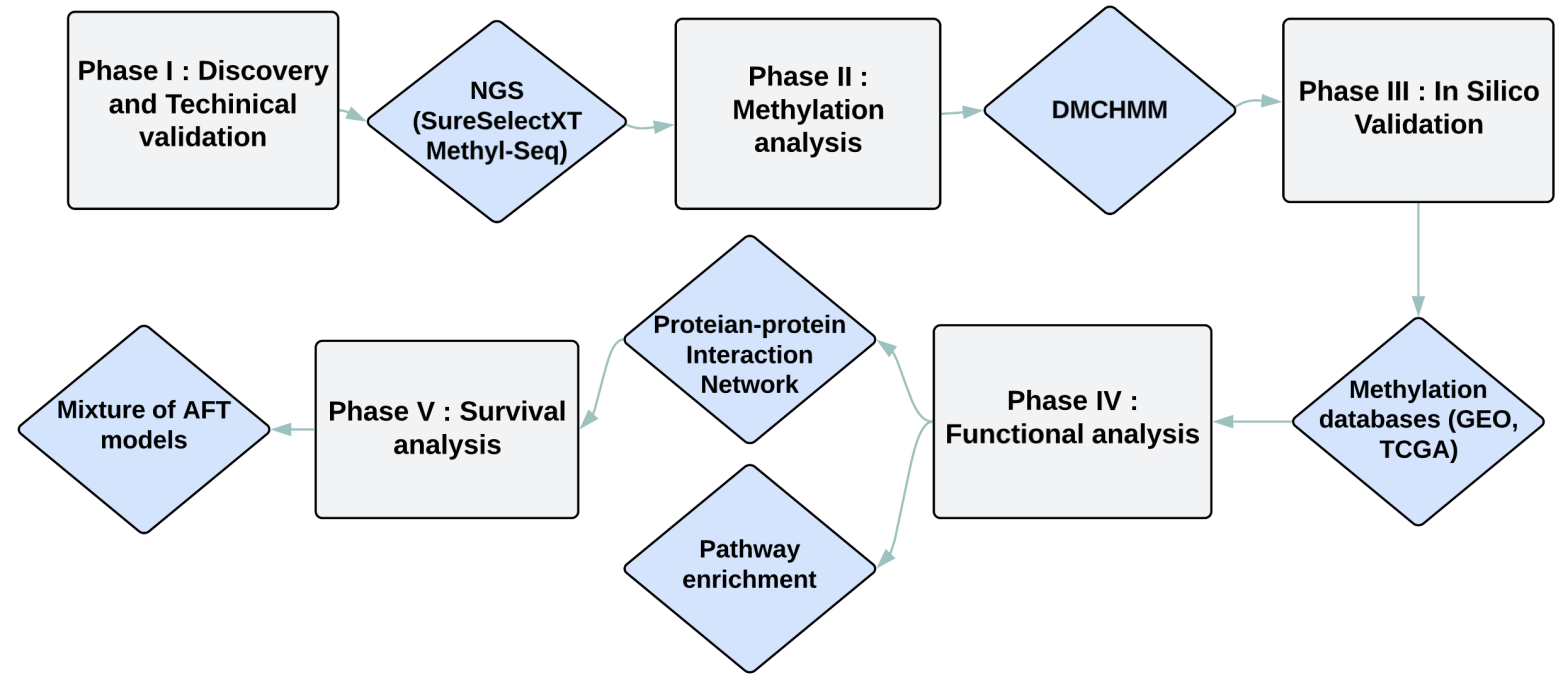
Study workflow for the analysis of CRC datasets.

In this section, we outline the data analysis process we followed to detect DMGs, hub genes, and their effects on the survival time and enriched pathways of CRC. Figure 1 depicts the flowchart of this process.

### Phase I (Pre-processing of discovery samples):

To identify methylation-based CRC biomarkers, information on 6 patients with adenocarcinoma of CRC and 6 normal males was obtained. Two groups were matched based on age, and family history of cancer^28^. This discovery dataset contains the methylation read counts and read-depth for each CpG site captured by SureSelectXT Human Methyl-Seq with 101 read length that generates 57 to 76 million Illumina sequencing reads per subject. Between 88.5% to 89.8% of sequenced reads were mapped to either strand of the human genome (GRCh37/19). The average number of times each CpG has been sequenced per sample was between 19X and 24X. The sequencing information of the subjects is presented in Table 1. Approximately, 68% of 19,530,818 CpG sites have missing information in at least one sample.

### Phase II (Identification of differentially methylated genes):

We utilized the DMCHMM pipeline^29^ to identify CpGs with differentially methylated patterns between CRC and normal discovery samples. We specifically did not remove any position with missing information or low read-depth. The missing information was imputed using DMCHMM via hidden Markov models^14^. Significant differentially methylated cytosines (DMCs) were selected based on the FDR threshold of 0.05. DMCs were aligned to the human reference genome (GRCh37/19) using the UCSC Genome Browser (https://genome.ucsc.edu). A gene whose promoter was mainly hypo- or hyper-methylated was classified as hypo- or hyper DMG, respectively.

### Phase III (Cross-platform validation):

To validate our result, several methylation profiles (GSE53051^30^, GSE77718^31^, GSE101764^13^, GSE42752,^32^ GSE48684^33^) were extracted from the Gene Expression Omnibus (GEO, https://www.ncbi.nlm.nih.gov/geo/). Of these datasets, a total of 212 CRC and 242 normal mucosa tissue samples were selected based on setup conditions to minimize the confounding effect of other variables. These datasets have provided valuable insights into the molecular alterations that occur in CRC, and their findings have implications for the diagnosis and treatment of this disease. For the analysis of methyl array profiles of validation sets, the GEO2R (http://www.ncbi.nlm.nih.gov/geo/geo2r/) web tool and the Limma R-package were used. A probe was considered differentially methylated if its adjusted p-value was less than 0.05, and the absolute of log_2_ of methylation fold change was greater or equal to 1. The differentially methylated probes were aligned to the human reference genome (GRCh37/19) using the FDb.InfiniumMethylation.hg19 package. In the last step, we compared the lists of DMGs based on the validation sets and our discovery samples to identify consistent hypo/hyper-methylated genes across different populations and platforms.

### Phase IV (Network construction and functional analysis):

In order to investigate the Protein–Protein-Interaction (PPI) network and module analysis, we utilized the ‘Search Tool for the Retrieval of Interacting Genes’ (STRING) database. We set the interaction score threshold to 0.4 to screen for high-confidence interactions and visualized the resulting network using the Cytoscape software (Version 3.9.1). Next, we employed the Molecular Complex Detection (MCODE) algorithm to uncover densely connected substructures within the network. The MCODE score must be greater than 3 and the minimum number of nodes must be 4. In order to identify key hub genes within the network, we used the cytoHubba plugin and considered the degree of centrality as a parameter.

To gain insight into the biological mechanisms that are driving CRC and prioritize identified DMGs, we performed functional and pathway enrichment analysis using DAVID (https://david.ncifcrf.gov/). Gene ontology (GO) terms and Kyoto Encyclopedia of Genes and Genomes (KEGG) pathways were considered significantly enriched if the p-values were less than 0.05 and the q-values were less than 0.1. The visualization of the identified GO terms and KEGG pathways were done with the clusterProfiler, pathfindR, and ShinyGO (http://bioinformatics.sdstate.edu/go/) packages.

### Phase V (Uncovering intangible heterogeneity of DMG effects on survival time):

**Figure 2.**
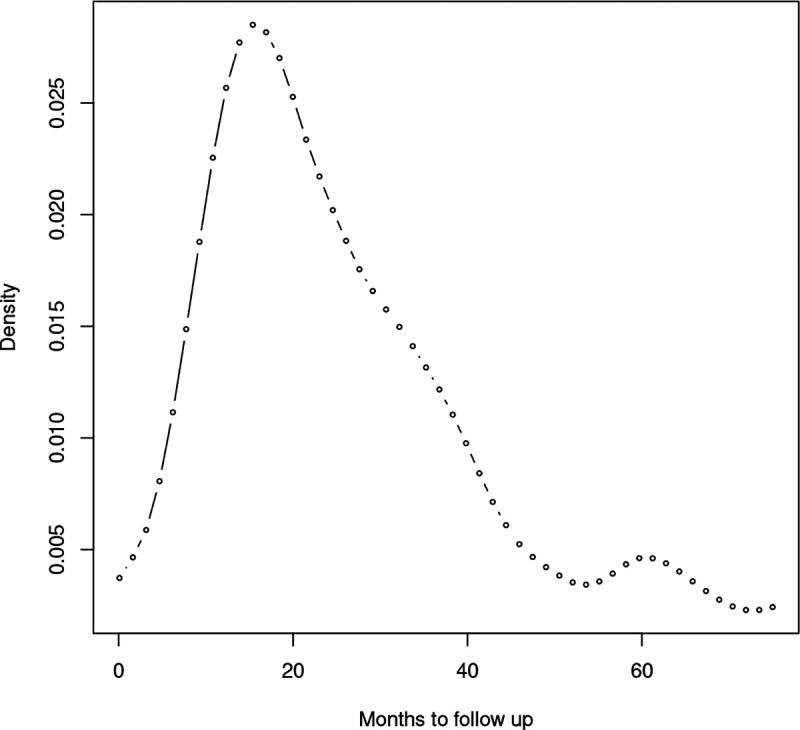
Density estimation of overall survival time (in months) in CRC patients.

To explore the relationship between identified DMGs and survival time, the DNA methylation profiles of 521 samples were obtained from The Cancer Genome Atlas (TCGA) network^34^. Complete information on clinical variables including days to follow-up and the status of the patient were analyzed. Preliminary analysis (Figure 2) and literature review confirmed the existence of heterogeneity (multiple sub-populations) in the distribution of survival times. Hence, we hypothesized that the effect of an identified DMG is different in each subpopulation. On the other hand, not all the DMGs have an effect on survival time (in each sub-population), which suggests that the underlying regression model is sparse. To account for sparsity as well as the heterogeneity of gene effects, the sparse estimation method in the finite mixture of AFT regression models^26^ was employed. Note that the response variable (survival time) is subject to right-censoring and the covariates are the centered, log-transformed average methylation of identified DMGs/hub genes.

It is common to screen the number of genes prior to analysis in case of a large number of identified genes. To this end, we applied a correlation-adjusted score method using the carSurv^27^ package. Next, we used the fmrs package^35^ to study the relationship between the survival time of the patients and the remaining DMGs using the smoothly clipped absolute deviations (SCAD) penalty^26^.

## 3 Results

### Differentially methylated cytosine detection:

**Figure 3.**
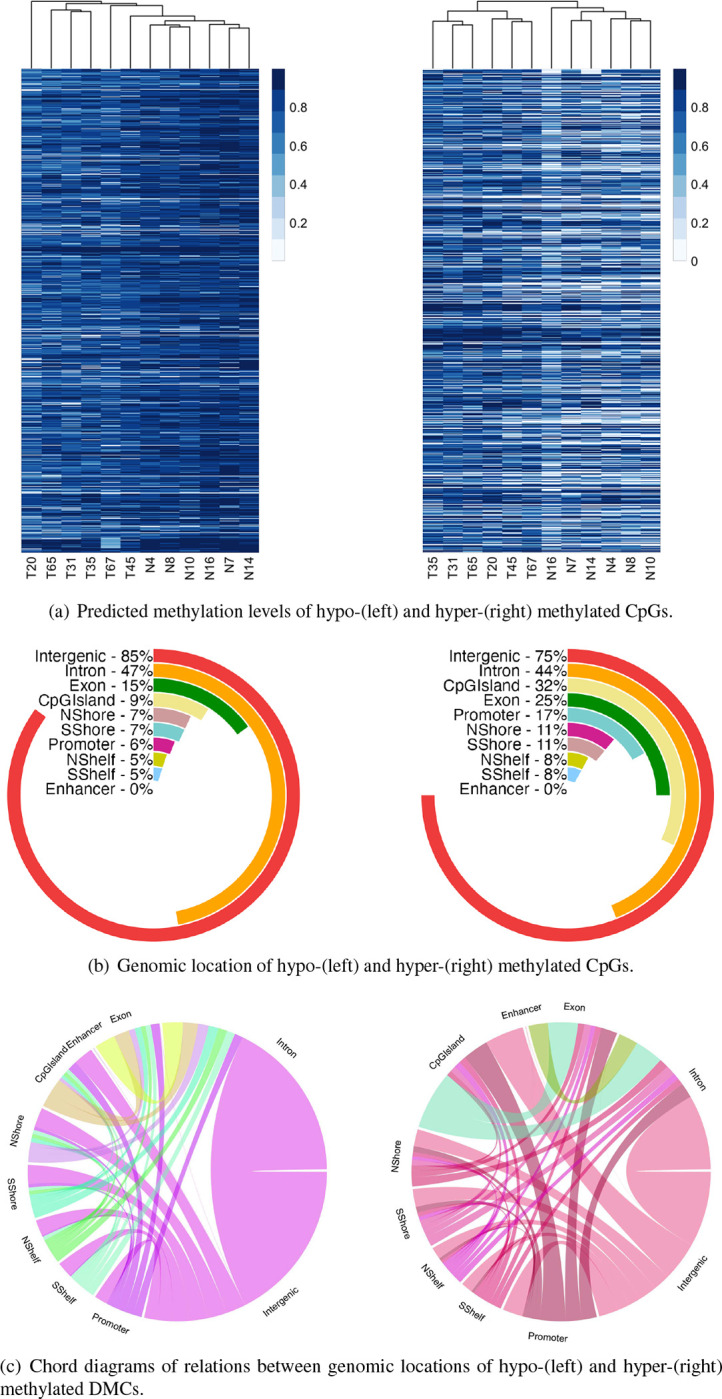
Genomic location of identified differentially methylated CpGs and their predicted levels in CRC (T) and normal (N) samples using DMCHMM. The hierarchical clustering of CRC and normal samples in the heatmaps is based on complete linkage.

We identified 2,691,019 DMCs between CRC and normal groups of the discovery dataset while adjusting for the potential confounding effect of smoking history or drug abuse. Of these identified DMCs, 1,985,557 positions were hypo-methylated and 705,462 CpGs were hyper-methylated in CRC vs normal samples. The heatmaps in Figure 3(a) indicate a clear clustering pattern between the CRC and normal samples based on the predicted methylation levels of DMCs.

To explore the genomic location of the DMCs, we analyzed their distribution across different regions and summarized the results in Figure 3(b). Intergenic regions were found to harbor the majority of the detected DMCs both in the hypo and hyper categories. Notably, we observed that 32% of hyper-methylated DMCs were located in CpG islands, while only 9% of hypo-methylated DMCs were located in these regions. Additionally, the regions with the highest percentage of hyper-methylated DMCs were identified in introns, exons, and CGI shores. Figure 3(c) gives a comprehensive overview of how hyper and hypo-methylated DMCs were distributed across different genomic regions. Our findings suggest that many DMCs in intergenic regions were expanded to intronic regions in both hypo and hyper-methylated categories.

Given the potential significance of promoter methylation in cancer development and progression, we focused our subsequent analysis on DMCs located on gene promoters, which encompassed 268,978 CpGs. These CpGs resided on 3,406 gene promoters, of which 1,394 were hyper-methylated and 2,012 were hypo-methylated. The list of DMGs is available as supplementary material.

### Robust DMGs in CRC:

**Figure 4.**
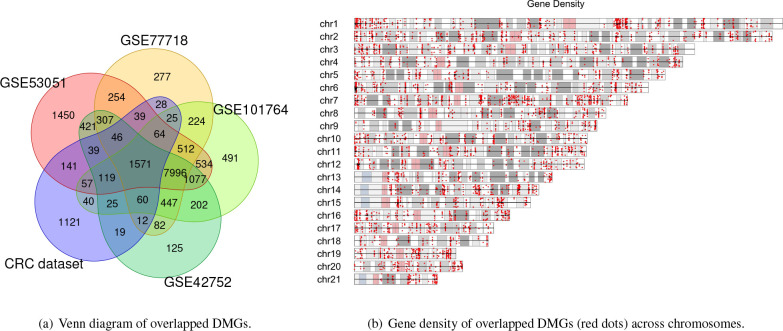
Summary of common identified DMG and their distribution.

To verify the robustness of identified DMGs, we performed a cross-platform procedure with DMGs identified in selected GEO datasets as depicted in Figure 4(a). The comparison revealed a total of 1571 overlapped DMGs that were consistently identified across multiple studies. As Figure 4(b) illustrated, the identified DMGs were spread almost evenly across different chromosomes, with chromosomes 1 and 7 having some dense regions of CRC-related DMGs. Within this set, 917 genes were hypo-methylated, and 654 genes were hyper-methylated. We focused our subsequent analysis on these identified DMGs to gain a deeper understanding of their role in CRC pathogenesis.

### GO enrichment KEGG pathway analysis:

**Figure 5.**
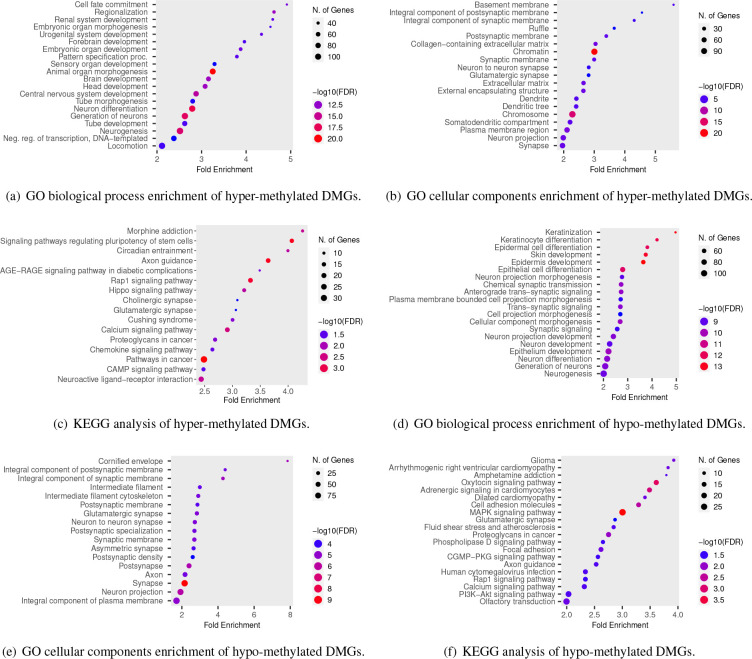
Enrichment analysis of commonly identified DMGs.

The analysis of robust DMGs in CRC utilizing the DAVID tool yielded a variety of enriched biological processes, molecular functions, and cellular components. Specifically, the hyper-methylated DMGs were found to be principally involved in ‘cell fate commitment’, ‘regionalization’, ‘embryonic organ morphogenesis’, ‘embryonic organ development’, ‘pattern specification process’, ‘animal organ morphogenesis’, ‘tube morphogenesis’, ‘tube development’, and ‘neurogenesis’ in the context of biological processes (Figure 5(a)). Enriched cellular components included ‘basement membrane’, ‘integral component of postsynaptic membrane’, and ‘Collagen-containing extracellular matrix’ (Figure 5(b)). Additionally, KEGG pathway analysis indicated that hyper-methylated DMGs were significantly enriched in several pathways, including ‘signaling pathways regulating pluripotency of stem cells’, ‘axon guidance’, ‘morphine addiction’, ‘rap1 signaling pathway’, ‘circadian entrainment’, and ‘pathways in cancer’ (Figure 5(c) and Table 2). Regarding biological processes, the hypo-methylated DMGs were found to be associated with a number of processes including ‘keratinization’, ‘keratinocyte differentiation’, ‘epidermal cell differentiation’, and ‘epithelial cell differentiation’ (Figure 5(d)). Furthermore, analysis of the cellular component pathway revealed that the hypo-methylated DMGs were most significantly enriched in the ‘cornified envelope’, ‘integral component of the synaptic membrane’, and ‘integral component of the postsynaptic membrane’. Notably, these cellular components demonstrated the highest FDR and fold enrichment (Figures 5(e)). Regarding molecular functions, the pathways with higher fold enrichment included ‘molecular transducer activity’, ‘signaling receptor activity’, and ‘transmembrane signaling receptor activity’. Notably, KEGG pathway analysis revealed that hypo-methylated DMGs were significantly enriched in several pathways, including the ‘oxytocin signaling pathway’, ‘glioma’, ‘adrenergic signaling in cardiomyocytes’, ‘MAPK signaling pathway’, ‘arrhythmogenic right ventricular cardiomyopathy’, and ‘cell adhesion molecules’ (Figures 5(f)). These results offer valuable insights into the potential mechanisms of DMGs in CRC and identify possible therapeutic targets for this disease. A comprehensive summary of the KEGG pathways of hyper-methylated DMGs can be found in Table 2.

### PPI network construction:

**Figure 6.**
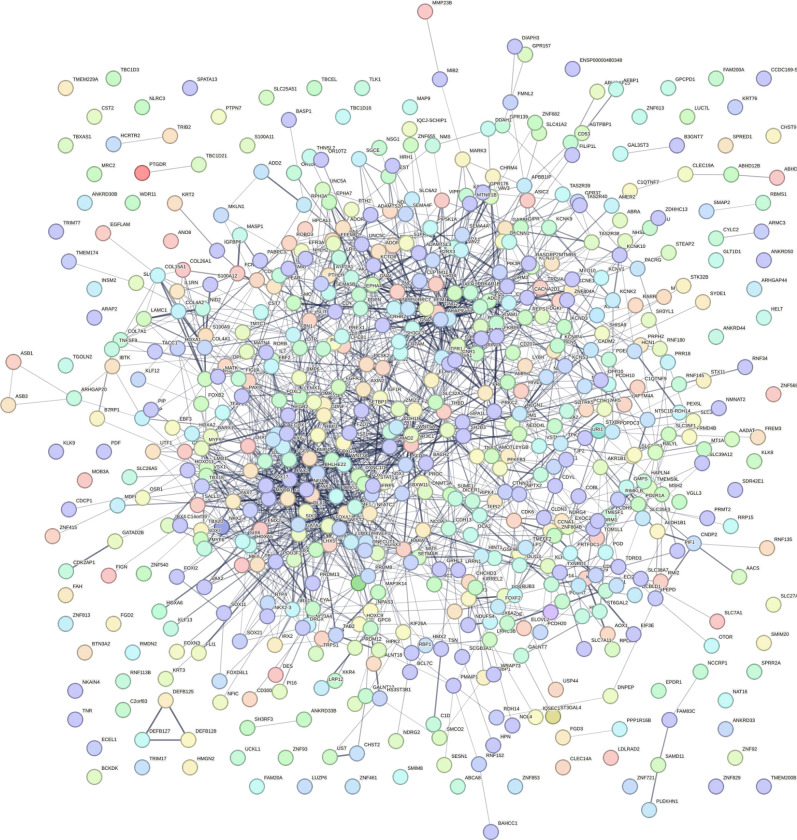
Protein–protein interaction network of hyper-methylated genes. Spots represent the proteins and lines show interactions.

**Figure 7.**
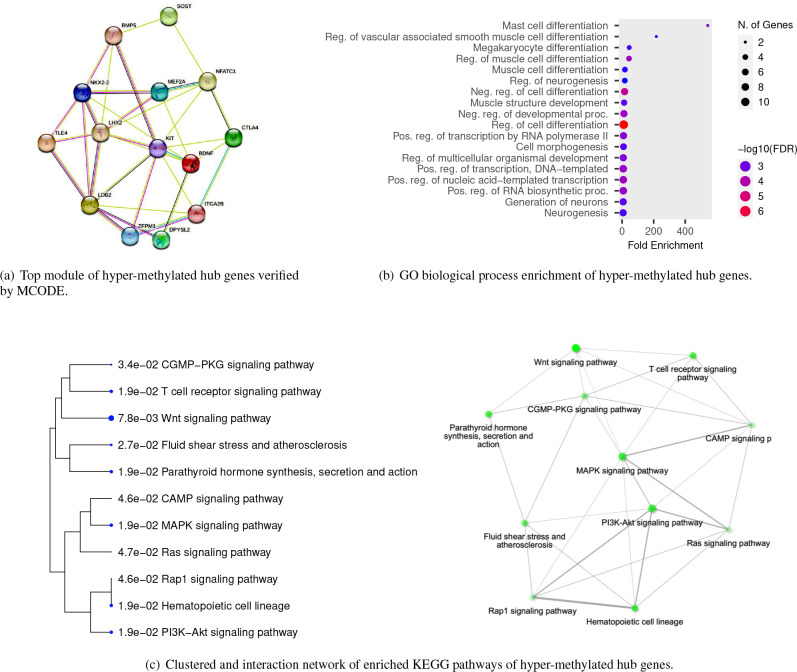
Bioinformatic analysis of hyper-methylated hub genes.

**Figure 8.**
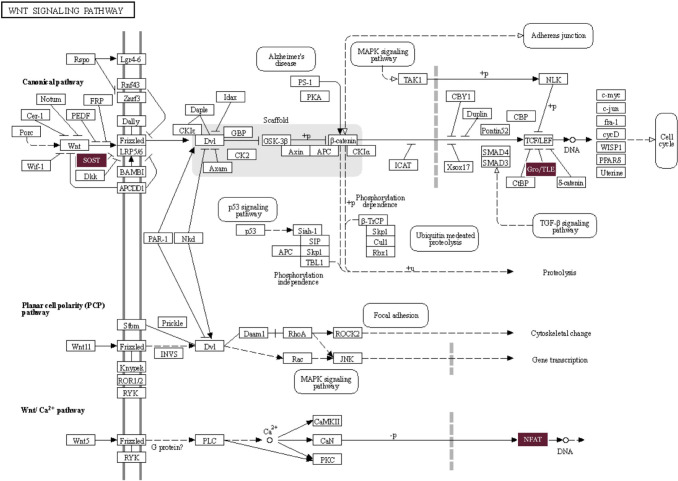
Wnt signaling pathway. The identified genes *SOST, Gro/TLE*, and *NFAT* are highlighted.

We ran a PPI network to further investigate the complex interactions between DMGs and find important hub proteins. A total of 606 PPI nodes of the hyper-methylated DMGs were constructed on the basis of the STRING database (Figure 6). The 16 node proteins, including *KIT, SEMA7A, BDNF, MEF2A, LDB2, GATA4, LHX2, SOST, CTLA4, NKX2–2, TLE4, BMP5, NFATC1, ZFPM1, DPYSL2,* and *ITGA2B* that showed a close interaction with other node proteins were chosen as hub genes (Figure 7(a)). The most important biological process and KEGG pathways of hub genes are shown in Figure 7(b)– 7(c). One important module was selected when the number of nodes is greater than 4. The key module demonstrated functions enriched in pathways such as Wnt signaling (Table 2 and Figure 8).

**Figure 9.**
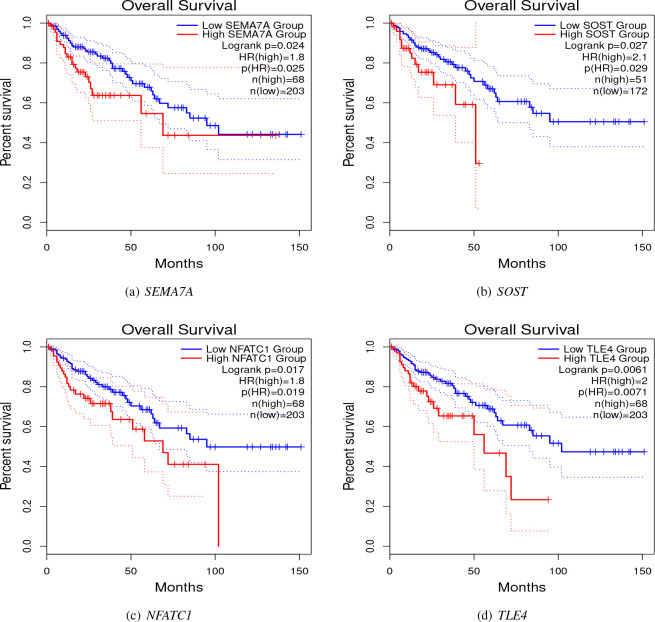
Overall survival of CRC patients stratified by their hub gene expression levels.

We performed a survival analysis using the TCGA-selected samples to investigate the association of selected hub genes with the survival time of CRC patients. Based on Figure 9(a)– 9(d), those patients with gene *SEMA7A* (*p* = 0.024), *SOST* (*p* = 0.027), *NFATC1* (*p* = 0.017), and *TLE4* (*p* = 0.0061) being upregulated, had a significantly lower probability of survival. However, this conclusion is based on univariate analysis, and the effect of other genes and the potential heterogeneity of DMG effects were ignored. We reanalyzed these data by accounting for the heterogeneity of DMG effects and obtained different results as follows.

### Intangible heterogeneity of DMG effects on survival time:

We studied the relationship between the average promoter methylation of the identified DMGs and the survival time subject to right-censoring by accounting for the heterogeneity of gene effects using an independent set of 521 TCGA CRC samples. To this end, we screened all the 1571 candidate DMGs using the correlation-adjusted regression survival scores to obtain the list of top candidate covariates. This process led to the selection of 95 highly correlated DMGs. These genes were also dysregulated in the TCGA samples. In addition, 4 hub genes that were related to the survival time of CRC patients were added to the list of covariates.

**Figure 10.**
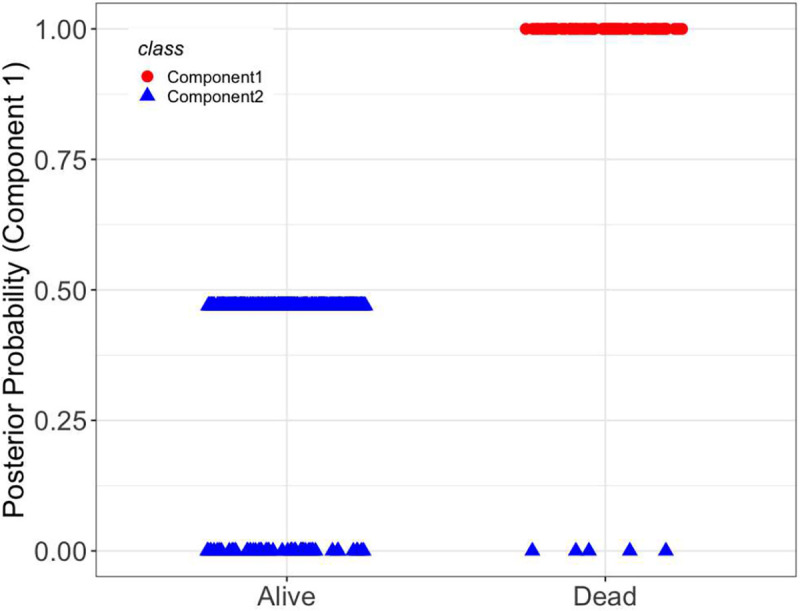
Posterior probability of CRC patients belonging to Component 1 separated for alive and deceased groups.

Our analysis yielded a two-component mixture of AFT regression model. The estimated gene effects on the survival time are given in Table 3. The result showed that 46% of the subjects were classified into Component 1, which is the most aggressive form of the disease. Figure 10 depicts the posterior probability of a subject belonging to Component 1. From this figure, we noticed that all living patients were classified into Component 2, which is the less aggressive form of the disease. A total of 83 and 18 DMGs were active in Components 1 and 2, respectively. Twelve genes including *HLA-F, MMP2, MT1A, RFPL4B, SIX6, ZFAT, BCKDK, AMOTL1, ADCY10, KCNK10, STAU2*, and *NOC4L* were not related to survival time in either of the components. These findings demonstrate the heterogeneity of DMG effects in CRC data and justify using a sparse mixture modeling rather than a univariate one. In addition, the DMGs with active promoters in Component 1 can be considered as biomarkers for CRC prognosis.

## 4 Discussion

Colorectal cancer is one of the deadliest cancers in the world. Given that early stages of CRC do not display symptoms, proactive screening is the only viable approach to identify the disease^36^. As DNA methylation changes are closely associated with cancer, their role in CRC biomarker detection in the early stages of cancer is of great importance. Although many CRC biomarkers have been detected in the literature, only a few are used in practice. Our findings resulted in identifying new biomarkers for CRC which can be used for diagnosis and prognosis.

We identified 1,571 DMGs most of which have been previously studied in the literature. Among them, *SEPT9, NDRG4, VIM, APC, SFRP1, SFRP4*, and *SFRP5*,^37^ are the most important CRC-related ones. We also explored CRC-related hub genes. Fourteen functional modules that may play important roles in the early detection of CRC were highlighted and the sub-network of hub genes *KIT, SEMA7A, BDNF, MEF2A, LDB2, GATA4, LHX2, SOST, CTLA4, NKX2–2, TLE4, BMP5, NFATC1, ZFPM1, DPYSL2*, and *ITGA2B* was extracted. These hub genes were flagged as potential diagnostic and therapeutic targets for CRC in our analysis.

In addition to the diagnostic role of our identified hub genes such as *NKX2–2, KIT, BNDF*, and *TLE4* in CRC and its sub-types^38–41^, their roles in increasing CRC risk, tumor progression, and targeted therapy have been investigated. For instance, *MEF2A*^42^ and *BMP5*^43^ increase the CRC risk. Up-regulation of the expression of *ITGB7* and *ITGA2B* has been found to be significantly associated with death by sodium butyrate-induced CRC organoids^44^. Moreover, some studies^45,46^ have shown effective treatments by targeting *CLT-4* and *LDB2n*.

There is a rich literature on the contribution of some of our identified hub genes in CRC and less evidence in support of some others such as *LHX2, ZFPM1*, and *DPYSL2*. For instance, the differences in tumor and corresponding adjacent benign tissues regarding *LHX* gene expressions have been investigated^47^. However, contrary to our findings, they did not find any statistical differences for *LHX2* and *LHX3* genes. Furthermore, the upregulation of *ZFPM1* was revealed in molecular high-risk patients with cytogenetically normal acute myeloid leukemia^48^, yet its diagnostic value in CRC has not fully been confirmed^49^. *SEMA7A* is also one of our selected hub genes that play a key role in several cancers including pancreatic, breast, and lung cancers^50–53^. However, there has been less attention on the role of *SEMA7A* in CRC. Further investigation is required on our flagged DMGs.

Although there are many mechanisms that drive CRC, only a handful of them has been discovered in past studies. As researchers continue to genotype large panels of CRC tumors, it can be expected that additional new pathways of CRC carcinogenesis will be revealed. *SOST*, an identified hub gene in our study, plays a vital role in inhibiting the Wnt signaling pathway by binding to the Wnt co-receptor, LRP5/6, and preventing its activation^54^. Therefore, decreased *SOST* expression could lead to an increase in Wnt signaling, promoting CRC cell proliferation, migration, and survival. Another identified hub gene is *TLE4* which is involved in the negative regulation of the canonical Wnt signaling pathway. Only a few investigations provided evidence of *TLE4* upregulation in CRC biopsies, partially through regulation of the JNK/c-Jun pathway^55^. Moreover, recent studies that focus on the NFAT signaling pathway showed a promising strategy for CRC treatment^56^.

Heterogeneity is one of the key features of genomic data. Specifically, there is evidence of the heterogeneity of DMG effects on the survival of CRC patients in the literature and in our dataset. The finite mixture of AFT regression model is a plausible method to uncover such intangible heterogeneity. Our analysis suggested a mixture of two-component mixture of AFT regression model in which patients were separated into two subgroups based on their vital status. In this model, almost all of the deceased patients were classified into the most aggressive form of the disease (Component 1). In Component 1, 83 DMGs including *NMNAT2, ZFP42, NPAS2, MYLK3, NUDT13, KIRREL3*, and *FKBP6* had an effect on the survival time of the patients. The relation between some of these DMGs and survival time has been previously reported^57^. On the other hand, there are a few discoveries regarding other genes. For instance, significantly higher expression of *NMNAT2* in CRC tissues compared to normal ones have been found, yet this gene was not a prognostic factor for overall survival^58^. Note that, while the hub genes *SOST, NFATC1*, and *TLE4* were associated with survival in the univariate Cox model, they were only associated with survival time in the most aggressive form of the disease in our study.

## Figures and Tables

**Table 1.** Summary statistics of methylation sequencing reads of discovery samples.

Sample	Total reads	Mapping Rate	Methylation (%)	Average Coverage	GC (%)

T65	76,723,684	88.50	47.70	24.15	27.04
N16	70,443,130	88.70	45.70	23.53	27.26
T20	67,394,464	88.90	44.70	19.58	27.03
N4	68,165,382	88.80	46.50	22.19	27.19
T31	61,789,306	89.00	46.90	21.69	26.92
N10	57,311,634	89.05	46.70	19.26	27.04
T35	79,004,644	88.90	46.10	24.43	27.11
N7	75,663,274	89.00	47.20	22.62	27.04
T45	64,188,480	89.00	47.40	21.22	27.06
N8	57,091,968	89.80	46.80	20.42	27.41
T67	61,203,576	89.30	44.30	20.77	27.17
N14	66,871,860	89.60	47.40	22.17	27.11

**Table 2.** KEGG pathway analysis of commonly identified hyper-methylated DMGs.

Enrichment FDR	nGenes	Pathway Genes	Fold Enrichment	Pathway	Matching proteins in network (labels)

0.0050	10	91	4.26	Morphine addiction	PDE8A, GNAS, SLC32A1, GABRA4, GNGT1, KCNJ3, ADORA1, ADCY1, PRKCB, GNG2
0.0004	15	143	4.07	Signaling pathways regulating pluripotency of stem cells	PAX6, FGFR1, LHX5, HOXA1, MYF5, WNT5A, ID2, BMP4, IGF1R, WNT3A, FZD1, FZD6, AXIN2, ONECUT1, SMAD2
0.0060	10	97	3.99	Circadian entrainment	GNAS, GNGT1, MTNR1B, ITPR1, KCNJ3, ADCY1, PRKCB, GRIN2A, PRKG1, GNG2
0.0004	17	181	3.64	Axon guidance	NEO1, PRKCZ, SEMA5B, NFATC2, CXCL12, UNC5A, WNT5A, EPHA4, SMO, EPHA7, SEMA4F, SEMA6D, SLIT2, ROBO3, UNC5C, SEMA4A, PLXNA4
0.0250	9	100	3.49	AGE-RAGE signaling pathway in diabetic complications	PRKCZ, STAT1, COL4A2, PLCD3, PRKCB, COL4A3, SMAD2, THBD, COL4A1
0.0006	18	210	3.32	Rap1 signaling pathway	PRKCZ, RASGRP2, APBB1IP, FGFR1, GNAS, FGF9, CNR1, VAV3, FGF5, IGF1R, ANGPT1, TIAM1, VAV2, ADCY1, PRKCB, ADORA2B, GRIN2A, SIPA1L1
0.0060	13	157	3.21	Hippo signaling pathway	CTNNA2, PRKCZ, FBXW11, TP73, WNT5A, ID2, BMP4, BMP6, WNT3A, FZD1, FZD6, AXIN2, SMAD2
0.0460	9	113	3.09	Cholinergic synapse	GNGT1, PIK3R5, ITPR1, KCNJ3, ADCY1, PRKCB, CHRM4, CHRM2, GNG2
0.0460	9	114	3.07	Glutamatergic synapse	GNAS, GNGT1, ITPR1, KCNJ3, ADCY1, PRKCB, GRIN2A, GNG2, GRM3
0.0180	12	155	3.00	Cushing syndrome	PDE8A, KCNK2, GNAS, CDK6, CRHR2, WNT5A, ITPR1, WNT3A, FZD1, ADCY1, FZD6, AXIN2
0.0030	18	240	2.91	Calcium signaling pathway	FGFR1, GNAS, FGF9, P2RX3, TACR1, FGF5, GNAL, ITPR1, PLCD3, ADCY1, PRKCB, GDNF, ADORA2B, OXTR, CHRM2, GRIN2A, ATP2A1, HRH1
0.0200	14	202	2.64	Chemokine signaling pathway	PRKCZ, RASGRP2, CXCL12, STAT1, PREX1, GNGT1, VAV3, PIK3R5, TIAM1, VAV2, ADCY1, PRKCB, GNG2
0.0500	11	166	2.56	Wnt signaling pathway	FBXW11, NFATC2, SFRP1, WNT5A, SFRP5, WNT3A, FZD1, SOX17, FZD6, PRKCB, AXIN2
0.0003	34	530	2.49	Pathways in cancer	CTNNA2, RASGRP2, FGFR1, GNAS, MSH2, FGF9, IL7, CDK6, CXCL12, WNT5A, STAT1, BMP4, GNGT1, SMO, RARA, CCNA1, COL4A2, LAMC1, FGF5, IGF1R, PMAIP1, WNT3A, FZD1, ADCY1, FZD6, PRKCB, AXIN2, COL4A3, SMAD2, GNG2, MITF, COL4A1, TXNRD1, NCOA4

**Table 3.** Estimated DMG effects in the two-component mixture of accelerated failure time regression model in the CRC data.

Gene	*β*_1_	*β*_2_	Gene	*β*_1_	*β*_2_	Gene	*β*_1_	*β*_2_

*NMI*	−27.2	0.0	*SIX6*	0.0	0.0	*FOXF2*	−12.1	0.0
*NCOA4*	−13.7	96804.5	*FOXP2*	12.6	−101775.9	*GIPR*	−19.0	0.0
*ANKMY1*	−32.6	0.0	*TNFSF9*	−14.7	0.0	*UCKL1*	−45.0	0.0
*ST6GAL2*	6.2	0.0	*CLDN3*	−2.1	21941.6	*AMOTL1*	0.0	0.0
*PSMG3*	−12.4	−28758.9	*DDX46*	40.0	0.0	*GMPS*	−6.8	0.0
*FAR2*	−21.6	0.0	*ZFAT*	0.0	0.0	*ADCY10*	0.0	0.0
*MPPED2*	−14.7	0.0	*OR5M1*	−6.0	0.0	*GPM6A*	−18.6	0.0
*GTF2IRD1*	−14.5	0.0	*PHACTR3*	6.3	0.0	*PFKP*	2.6	0.0
*FKBP6*	−11.9	0.0	*KRTAP13-4*	4.7	−15847.1	*C14orf39*	2.4	−15364.8
*SNORD109B*	−6.5	0.0	*LOC400940*	−6.6	70576.5	*KCNK10*	0.0	0.0
*HLA-F*	0.0	0.0	*LRTM1*	−13.4	−50609.5	*STK32B*	18.4	0.0
*AKAP9*	7.1	0.0	*NPAS2*	125.0	0.0	*IL1A*	13.3	0.0
*SEMA4F*	−21.3	0.0	*AXIN2*	24.3	0.0	*KRTAP20-1*	5.0	0.0
*RPL23P8*	18.1	0.0	*NKX2-3*	0.0	−13689.5	*KIRREL2*	−13.1	0.0
*CHI3L1*	4.6	0.0	*NT5M*	18.8	0.0	*C1D*	−28.8	0.0
*NCAN*	3.7	151828.5	*MECOM*	44.5	0.0	*EGR2*	54.7	0.0
*CLEC5A*	−10.4	0.0	*LUZP6*	−73.9	0.0	*PDF*	−1.4	8045.7
*TRPS1*	−16.7	0.0	*FLJ16779*	0.0	−87806.8	*KCNQ3*	23.4	0.0
*CMKLR1*	18.1	0.0	*SLC25A24*	−6.0	−77923.0	*CCR5*	−20.3	0.0
*GABRA4*	−6.2	0.0	*C1QTNF7*	−10.6	0.0	*COL4A3*	0.0	34903.1
*OR5AS1*	−39.6	0.0	*MTNR1B*	11.7	0.0	*TFAP2C*	7.9	0.0
*MMP2*	0.0	0.0	*NMNAT2*	−12.0	0.0	*GNG2*	7.1	0.0
*AKAP12*	6.6	0.0	*BCKDK*	0.0	0.0	*OC90*	0.8	80377.8
*PSD2*	5.4	−82538.6	*ZFP42*	−13.8	0.0	*LHFPL2*	21.5	0.0
*FGFR1*	14.0	0.0	*CALB1*	−5.9	0.0	*STAU2*	0.0	0.0
*KIRREL3*	−10.3	0.0	*TCHH*	−17.8	0.0	*OLFM3*	10.3	0.0
*HECA*	−6.8	0.0	*MAPT*	−14.1	0.0	*SLTM*	−133.5	0.0
*MT1A*	0.0	125763.0	*SYDE1*	4.2	−364254.7	*NOC4L*	0.0	0.0
*NUDT13*	−7.3	0.0	*RNASE3*	7.0	0.0	*CNDP2*	0.0	0.0
*STON1-GTF2A1L*	−21.3	0.0	*PLCD3*	58.7	0.0	*NFATC1*	−20.3	0.0
*LBP*	−7.7	0.0	*MAP1LC3A*	5.8	0.0	*SEMA7A*	21.4	0.0
*MYLK3*	21.9	0.0	*CROCC*	18.2	0.0	*SOST*	−2.5	0.0
*RFPL4B*	0.0	0.0	*OPCML*	21.4	0.0	*TLE4*	−7.2	0.0

## Data Availability

In this study methylation profiling datasets with accession numbers GSE53051, GSE77718, GSE101764, GSE42752, and GSE48684 were obtained from Gene Expression Omnibus (GEO, https://www.ncbi.nlm.nih.gov/geo/), of the National Center for Biotechnology Information (NCBI). Additional DNA methylation datasets and expression profiles of CRC patients (TCGA-COAD, TCGA-READ, TCGA-SARC projects) were obtained from The Cancer Genome Atlas (TCGA, https://www.cancer.gov/ccg/research/genome-sequencing/tcga), of the National Cancer Institute (NCI). Our SureSelectXT Human Methyl-Seq dataset on methylation profiles of 6 patients with adenocarcinoma of CRC and 6 normal males is obtained from ‘Reza Radiotherapy and Oncology Center’ in Iran and is available upon request.
